# Case report: Satralizumab as an adjunctive therapy for AQP-4 antibody and MOG antibody dual-negative optic neuritis in a third-trimester pregnancy case

**DOI:** 10.3389/fmed.2024.1514687

**Published:** 2024-12-02

**Authors:** Chuanbin Sun, Zhe Liu

**Affiliations:** ^1^Eye Center, Second Affiliated Hospital of Zhejiang University School of Medicine, Hangzhou, China; ^2^Department of Ophthalmology, Zhejiang Provincial People’s Hospital, People’s Hospital of Hangzhou Medical College, Hangzhou, China

**Keywords:** optic neuritis, pregnancy, methylprednisolone pulse therapy, immunoglobulin therapy, satralizumab

## Abstract

The treatment of demyelinating optic neuritis (DON) in pregnant patients is challenging, especially when there is poor or no response to intravenous methylprednisolone pulse (IVMP) therapy or adjunctive treatments such as intravenous immunoglobulin (IVIG) therapy. We herein report a case of a 28-year-old pregnant woman who experienced sequential severe vision loss in both eyes. She presented to a local hospital with the main complaint of sudden, painless vision loss in the left eye and was diagnosed with DON in the left eye. However, she did not receive orbital MRI or IVMP therapy due to safety concerns. Upon admission to our hospital, her visual acuity was 20/30 in the right eye and there was no light perception in the left eye. Her right eye vision deteriorated rapidly, declining to 20/1,000 one day after the admission. The ophthalmic examination revealed a normal anterior segment and a swollen optic disk in the right eye and a dilated pupil with a relative afferent pupillary defect and a swollen optic disk in the left eye. The serological tests for common pathogens, including the aquaporin-4 antibody (AQP-4 Ab), myelin oligodendrocyte glycoprotein antibody (MOG-Ab), and other common autoantibodies, were all negative. The patient was clinically diagnosed with DON in both eyes and received 7 days of IVMP therapy and 4 days of IVIG therapy, but showed no visual improvement. A three-dose regimen of satralizumab 120 mg was then administered subcutaneously during the acute stage of DON, in combination with a slowly tapered oral methylprednisolone regimen. Moreover, 2 months after the first injection of satralizumab, the patient naturally gave birth to a healthy female infant weighing 2,305 g at 36 weeks and 1 day of gestation. Her visual acuity improved to 20/500 in both eyes and slightly increased to 20/320 in both eyes 2 months later. Her visual acuity remained stable during subsequent follow-up visits. The infant was fed formula milk powder and developed normally. No systemic or ocular side effects related to satralizumab therapy were observed in the patient or her fetus during the 9-month follow-up. Our findings in this case suggest that satralizumab may be a safe and efficient adjunctive therapy for pregnant patients with DON who poorly respond to IVMP and IVIG therapy, even in cases of dual-negative AQP-4 Ab and MOG-Ab.

## Introduction

Demyelinating optic neuritis (DON) is a rare but sight-threatening ophthalmic condition (1, 2). DON usually occurs idiopathically but can also be accompanied by the aquaporin-4 antibody (AQP-4 Ab), myelin oligodendrocyte glycoprotein antibody (MOG-Ab), or other autoantibodies, such as antinuclear antibodies (1–3). Considering that DON predominantly affects women of childbearing age, pregnancy is not uncommon in female patients with DON (1, 3–9).

Although the majority of pregnant patients with DON show a good response to intravenous methylprednisolone pulse (IVMP) therapy, few patients respond poorly and require adjunctive therapies such as plasma exchange, immunoadsorption, or intravenous immunoglobulin (IVIG) therapy (1–7). However, some pregnant patients with DON still show a poor or no response to these adjunctive therapies (7), posing a challenge for neuro-ophthalmologists regarding the next course of action. Herein, we present a case report on the use of satralizumab as an adjunctive therapy in a third-trimester pregnant patient with DON who poorly responded to IVMP and IVIG therapies.

## Case report

A 28-year-old woman presented to a local hospital with the main complaint of sudden, painless vision loss in the left eye. The initial ophthalmic examination revealed no light perception, a dilated pupil, and a swollen optic disk in her left eye, while her right eye appeared normal. Her medical history was unremarkable, except for a confirmed pregnancy of 30 weeks. The immediate serum screening tests for common pathogens, such as *Treponema pallidum, Tubercle bacillus*, hepatitis B virus, hepatitis C virus, human immunodeficiency virus, cytomegalovirus, rubella virus, herpes viruses, and toxoplasma, were all negative.

The patient was diagnosed with DON in the left eye. However, cranial or orbital MRI examination and intravenous methylprednisolone pulse therapy were not performed due to safety concerns. Instead, a sustained-release dexamethasone implant (Ozurdex, Allergan Pharmaceuticals, Ireland) was injected into the vitreous of her left eye. After 5 days, the patient complained of a constricted visual field in her right eye and no visual improvement in her left eye.

**Figure 1:**
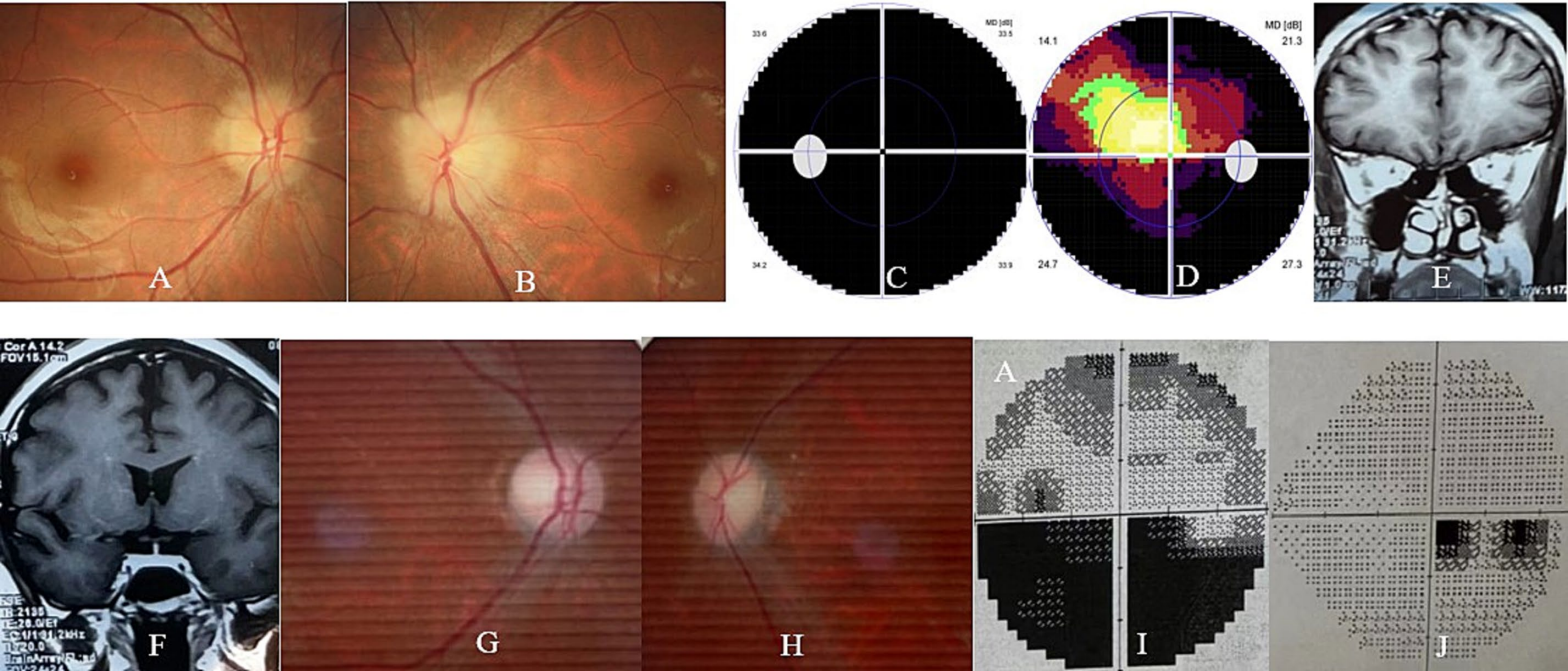
Ophthalmic and orbital MRI examination of the patient. At presentation, the ophthalmic examination revealed bilateral optic disk swelling (A,B) and the visual field test showed diffuse depression (C) and a tubular visual field (D) in the left and right eye, respectively. At the 2-month follow-up, the orbital MRI examination revealed slightly thin bilateral optic nerves (E) and chiasm (F) in both eyes. At the 6-month follow-up, the ophthalmic examination revealed bilateral temporal optic disk pallor (G,H), and the visual field test showed an inferior hemifield defect (I) and a centrocecal scotoma (J) in the left and right eye, respectively.

The patient was then referred to our neuro-ophthalmology clinic for further consultation. Upon admission to our hospital, her visual acuity was 20/30 in the right eye and there was no light perception in the left eye. The ophthalmic examination revealed a normal anterior segment and a swollen optic disk in the right eye (Figure 1A) and a dilated pupil with a relative afferent pupillary defect and a swollen optic disk in the left eye (Figure 1B). The visual field test showed diffuse depression and a tubular visual field in the left and right eye, respectively (Figure 1C,D). The serological tests for erythrocyte sedimentation rate, C-reactive protein, and common autoantibodies, including antinuclear antibodies, antiphospholipid antibodies, and antineutrophil cytoplasmic antibodies, were all negative. The commercial serological tests for AQP-4 Ab and MOG-Ab using a cell-based indirect dual-immunofluorescence assay were negative. Unfortunately, orbital MRI examination was also declined by the radiologist at our hospital due to safety concerns.

The patient was then diagnosed with DON in both eyes based on the clinical evidence, although orbital and sellar masses, or even infiltrative optic neuropathy, could not be excluded due to the lack of information on MRI examination. Unfortunately, her right eye vision continued to deteriorate to 20/500, and her left eye vision still had no light perception 1 day after the admission. After detailed discussions with the patient and her family about the severity of her ocular condition, as well as the necessity and risks of IVMP, the patient decided to proceed with IVMP therapy. Hence, an intravenous injection of methylprednisolone 500 mg twice per day for 3 days was administered. However, her vision in the right eye further decreased to 20/1,000, despite 3 days of IVMP therapy. As a result, IVIG (400 mg/kg once per day for 4 days) combined with intravenous methylprednisolone (250 mg twice per day for 4 days) were further administered. Unfortunately, there was still no visual improvement in both eyes. Plasma exchange or immunoadsorption was then highly recommended to preserve her vision, but the patient declined the treatment.

After further detailed discussions with the patient and her family about the severity of her current condition and other alternative adjunctive therapies—especially the off-label use of monoclonal antibody therapies such as rituximab, ofatumumab, inebilizumab, and satralizumab along with their respective advantages and disadvantages—the patient finally chose satralizumab as an adjunctive therapy. Then, 7 days after her admission (2 weeks after the initial onset of DON), the patient received the first dose of satralizumab (120 mg subcutaneously) and was discharged with a prescription of oral methylprednisolone 48 mg once per day. The oral methylprednisolone was then gradually tapered and finally discontinued after 6 months. The second and third doses of satralizumab were administered without complications 2 and 4 weeks after the initial injection, respectively. The periodic physical and ultrasound examinations of the patient and fetus were unremarkable before and after the steroid and satralizumab therapies during admission and follow-up.

Two months after the first injection of satralizumab, the patient naturally delivered a healthy female infant weighing 2,305 g at 36 weeks and 1 day of gestation. Her visual acuity improved to 20/500 in both eyes and then slightly increased to 20/320 in both eyes 2 months later. Her visual acuity remained stable during subsequent follow-up. Her infant was fed formula milk powder and has developed normally to date, i.e., 7 months after the childbirth.

Orbital MRI was immediately performed at a local hospital 2 weeks after her delivery. Unfortunately, the quality of the MRI images was suboptimal as a fat-suppressed sequence was not used for the optic nerve examination. However, slightly thin bilateral optic nerves and chiasm, without any orbital or sellar mass, could be clearly observed in her MRI images (Figure 1E,F).

At the final follow-up (9 months after the onset of DON), her visual acuity remained 20/320 in both eyes. The ophthalmic examination revealed a pale optic disk in both eyes (Figure 1G,H), and the visual field test revealed an inferior hemifield defect and a centrocecal scotoma in the left and right eye, respectively (Figure 1I,J). The second serum test for AQP-4 Ab and MOG-Ab using a cell-based assay performed at the other hospital was still negative. There was no relapse of DON, and no systemic or ocular side effects related to satralizumab therapy were observed in the patient and fetus/infant during the 9-month follow-up. The patient and her infant continue to receive follow-up care, but no steroid or satralizumab therapy is currently being administered.

## Discussion

DON in pregnant patients usually occurs in isolation but can also present as an ocular manifestation of neuromyelitis optica spectrum disease (NMOSD), MOG-Ab-related disease, or multiple sclerosis (1, 3–6). IVMP therapy is the preferred treatment for DON in pregnant patients, and it usually results in good visual recovery (1, 3–6). For pregnant patients with DON who poorly respond to, who are unsuitable for, or who are unwilling to undergo IVMP therapy, adjunctive therapies such as plasma exchange, immunoadsorption, or IVIG are highly recommended to preserve visual function (1, 4–6).

However, it would present a great challenge for neuro-ophthalmologists if some pregnant patients with DON are unresponsive to, unavailable for, or even unwilling to undergo the abovementioned adjunctive therapies. In fact, plasma exchange and immunoadsorption therapy may sometimes be unavailable due to plasma shortages, prohibitive costs, or allergic reactions. Moreover, IVIG therapy may help alleviate symptoms and prevent disease progression, but its efficacy in improving visual function is still under discussion (6, 7, 10). In this circumstance, monoclonal antibodies such as rituximab and satralizumab are alternative adjunctive therapies for refractory DON during pregnancy. However, we do not suggest that an intravitreal injection of a sustained-release dexamethasone implant, as performed in this case at a local hospital, is an appropriate treatment choice for DON, regardless of whether it is accompanied by pregnancy.

Safety is the most important concern when selecting therapies for pregnant patients with DON (11–14). Methylprednisolone and prednisone have been proven to be safe for both pregnant women and their fetuses. However, mycophenolate mofetil and methotrexate are contraindicated in pregnant patients due to their teratogenic potential (11). Azathioprine and rituximab are considered relatively safe during pregnancy and lactation since their side effects have been rarely reported in the literature. Previous studies have revealed that azathioprine may occasionally lead to miscarriage, infection, anemia, neutropenia, lymphopenia, or even congenital abnormalities such as ectrodactyly (1, 8, 9, 11–15). In contrast, rituximab may rarely cause premature labor, infections, lymphopenia, neutropenia, or congenital abnormalities (9–13, 16, 17). Currently, there is still a lack of safety data regarding the use of inebilizumab in pregnant patients with DON. Given that inebilizumab is mechanistically and pharmacologically very similar to rituximab, its safety profile during pregnancy may also be quite similar (11, 12). Current clinical experience indicates that satralizumab and tocilizumab appear to have favorable safety profiles during pregnancy and lactation. As a monoclonal antibody specific to the interleukin-6 (IL-6) receptor, IgG2-formed satralizumab, compared to IgG1-formed tocilizumab, may be much safer for DON cases during pregnancy and lactation (11, 18). A case report revealed that satralizumab has a longer plasma half-life and lower placental passage and transfer compared to tocilizumab. Hence, satralizumab may be safely used during pregnancy and breastfeeding. However, transient neutropenia, particularly severe febrile neutropenia, although rare, has been reported in previous studies (11, 12, 18). Premature labor and low birth weight in the newborn were also observed in our case. Fortunately, the infant’s physical examination at birth was normal in all aspects, and the infant developed normally during the follow-up. This suggests that satralizumab is safe for use in DON cases during pregnancy, as indicated by this study.

IL-6 is one of the most important pro-inflammatory cytokines and is considered to play an important role in the pathogenesis of NMOSD and MOG-AD. Previous studies have shown that IL-6 levels are significantly increased during the onset or relapse phase of some MOG-AD and NMOSD cases. In addition, anti-IL-6 receptor antibodies such as satralizumab have demonstrated good therapeutic effects in AQP4-Ab-positive NMOSD and MOG-AD (7). However, the efficacy of satralizumab therapy in AQP4-Ab-negative NMOSD cases is still under discussion (12, 19). A phase 3 clinical trial revealed that satralizumab monotherapy did not decrease the relapse rate in AQP4-Ab-negative NMOSD cases (12, 19).

To the best of our knowledge, this is the first case report of satralizumab therapy for AQP-4 Ab and MOG-Ab dual-negative isolated DON during pregnancy. Our findings, in this case, suggest that satralizumab may be a safe and effective adjunctive therapy for pregnant patients with DON who poorly respond to IVMP and IVIG, even when the DON is AQP-4 Ab and MOG-Ab dual-negative. However, a large-scale randomized control trial is needed to further evaluate whether the visual improvement in refractory DON, as in our case, is due to adjunctive satralizumab therapy or simply a delayed therapeutic effect of steroid treatment.

## Data Availability

The original contributions presented in the study are included in the article/Supplementary material, further inquiries can be directed to the corresponding author.
